# The Correlation Between Busulfan Exposure and Clinical Outcomes in Chinese Pediatric Patients: A Population Pharmacokinetic Study

**DOI:** 10.3389/fphar.2022.905879

**Published:** 2022-06-16

**Authors:** Xiaohuan Du, Chenrong Huang, Ling Xue, Zheng Jiao, Min Zhu, Jie Li, Jun Lu, Peifang Xiao, Xuemei Zhou, Chenmei Mao, Zengyan Zhu, Ji Dong, Xiaoxue Liu, Zhiyao Chen, Shichao Zhang, Yiduo Ding, Shaoyan Hu, Liyan Miao

**Affiliations:** ^1^ Department of Pharmacy, The First Affiliated Hospital of Soochow University, Suzhou, China; ^2^ Department of Pharmacy, The Children’s Hospital of Soochow University, Suzhou, China; ^3^ Institute for Interdisciplinary Drug Research and Translational Sciences, College of Pharmaceutical Science, Soochow University, Suzhou, China; ^4^ Department of Pharmacology, University of the Basque Country (UPV/EHU), Leioa, Spain; ^5^ Department of Pharmacy, Shanghai Chest Hospital, Shanghai Jiao Tong University, Shanghai, China; ^6^ Department of Hematology and Oncology, The Children’s Hospital of Soochow University, Suzhou, China; ^7^ National Clinical Research Center for Hematologic Diseases, The First Affiliated Hospital of Soochow University, Suzhou, China

**Keywords:** event-free survival, busulfan exposure, population pharmacokinetics, pediatric patients, hematopoietic stem cell transplantation

## Abstract

**Aims:** The aims of the study were to 1) establish a population pharmacokinetic (Pop-PK) model for busulfan in Chinese pediatric patients undergoing hematopoietic stem cell transplantation (HSCT) and then estimate busulfan exposure and 2) explore the association between busulfan exposure and clinical outcomes.

**Methods:** A total of 128 patients with 467 busulfan concentrations were obtained for Pop-PK modeling using nonlinear mixed effect model (NONMEM) software. Sixty-three patients who received the 16-dose busulfan conditioning regimen were enrolled to explore the correlations between clinical outcomes and the busulfan area under the concentration–time curve (AUC) using the Cox proportional hazards regression model, Kaplan–Meier method and logistic regression.

**Results:** The typical values for clearance (CL) and distribution volume (V) of busulfan were 7.71 L h^−1^ and 42.4 L, respectively. The allometric normal fat mass (NFM) and maturation function (F*mat*) can be used to describe the variability in CL, and the fat-free mass (FFM) can be used to describe the variability in V. Patients with AUCs of 950–1,600 µM × min had 83.7% (95% CI: 73.3–95.5) event-free survival (EFS) compared with 55.0% (95% CI: 37.0–81.8) for patients with low or high exposure (*p* = 0.024). The logistic regression analysis results showed no association between transplant-related toxicities and the busulfan AUC (*p* > 0.05).

**Conclusions:** The variability in busulfan CL was related to the NFM and F*mat*, while busulfan V was related to the FFM. Preliminary analysis results suggested that a busulfan AUC of 950–1,600 µM × min was associated with better EFS in children receiving the 16-dose busulfan regimen.

## Introduction

Hematopoietic stem cell transplantation (HSCT) effectively treats many life-threatening malignant and nonmalignant diseases in pediatric patients (McCune and Holmberg, 2009). Busulfan is a bifunctional alkylating agent widely used in conditioning regimens in combination with cyclophosphamide (CTX), fludarabine and other chemotherapeutic drugs applied before HSCT. Busulfan is known for high variability in intra- and interindividual pharmacokinetics (PK) and pharmacodynamics (PD), especially in children (Nguyen et al., 2004; Booth et al., 2007; Bartelink et al., 2012; Savic et al., 2013; Long-Boyle et al., 2015; Marsit et al., 2020).

To identify relevant relationships between busulfan PK and potential covariates, population PK (Pop-PK) models have been established for different populations. Based on the Pop-PK model, weight-based initial dosing strategies were proposed by the United States FDA, and the European Medicines Agency (EMA) created in children (Nguyen et al., 2004; Booth et al., 2007). Except for body weight (Nguyen et al., 2004; Booth et al., 2007; Bartelink et al., 2012), other Pop-PK models have shown that age (Savic et al., 2013; McCune et al., 2014), body surface area (BSA) (Trame et al., 2011; Wang et al., 2015; Rhee et al., 2017), body composition (McCune et al., 2014; van Hoogdalem et al., 2020), coadministered drugs (such as fludarabine) (Ishiwata et al., 2018) were associated with busulfan clearance (CL). Notably, the influence of genetic polymorphisms in glutathione S-transferase (GST) enzymes, which mediate the metabolism of busulfan conjugation with glutathione, affect the degree of busulfan metabolism and may cause variability in busulfan PK (Yin et al., 2015; Ansari et al., 2017; Nava et al., 2017; Kim et al., 2019). *GSTA1*, the predominant enzyme in busulfan metabolism, was first introduced as one of the covariates into a Pop-PK model in children and adolescents (Nava et al., 2018). Recently, Yuan et al. (2021) also showed a lower CL of busulfan in patients with the *GSTA1* mutant genotype than in those with the wild-type in a Chinese pediatric population (Yuan et al., 2021).

However, the pediatric patients with these alternative Pop-PK models and dosing nomograms were mainly Caucasian (Nguyen et al., 2004; Booth et al., 2007; Trame et al., 2011; McCune et al., 2014; Nava et al., 2018; van Hoogdalem et al., 2020), Japanese (Nakamura et al., 2008; Ishiwata et al., 2018), and Korean (Rhee et al., 2017) populations. As we know, there are a few Pop-PK studies on busulfan in Chinese adults (Wu et al., 2017; Huang et al., 2019; Sun et al., 2020), and there are only two Pop-PK studies on busulfan in Chinese children (Yuan et al., 2021; Huang et al., 2022). A systemic external evaluation of 11 published models on busulfan using data from Chinese pediatric patients by Huang et al. (2022) showed that the model developed only from the Korean population (Rhee et al., 2017) was suitable for Chinese HSCT pediatric patients (Huang et al., 2022). Therefore, due to the differences in body size, dietary habits and genetic background of the populations included in different Pop-PK studies, continued Pop-PK studies in the Chinese pediatric population are warranted to establish a platform for individualized busulfan administration.

It is widely understood that systemic exposure to busulfan measured as the area under the concentration time curve (AUC) or average steady-state concentration (Css) has a certain correlation with clinical outcomes (Bartelink et al., 2016; Palmer et al., 2016). Subexposure (< AUC 900 µM × min or Css 600 ng ml^−1^) results in higher rates of failure, graft rejection and disease relapse (Slattery et al., 1995; McCune et al., 2002; Bartelink et al., 2009). However, overexposure (>AUC 1500 µM × min or Css 900 ng ml^−1^) is associated with an increased risk of transplant-related toxicities (TRTs), such as veno-occlusive disease (VOD), acute graft-versus-host disease (aGVHD) and transplantation-related mortality (Grochow et al., 1989; Dix et al., 1996; Ljungman et al., 1997; Andersson et al., 2002). In children, the target exposures recommended by the FDA and EMA dosage regimens are 900–1,350 ± 5% µM × min and 900–1,500 µM × min for 6-hourly intravenous (IV) busulfan administration, respectively (Palmer et al., 2016). Nevertheless, studies on the relationship between busulfan exposure and clinical outcomes in Chinese populations are still scarce (Yin et al., 2015), especially among children (Shao et al., 2022).

Hence, the aims of the current study were 1) to characterize the PK of IV busulfan in Chinese children using Pop-PK analysis; 2) to evaluate the associations between busulfan exposure (expressed as AUC) and clinical outcomes in patients receiving a 16-dose busulfan myeloablative regimen (four times daily for four consecutive days), and preliminarily explore the optimum busulfan therapeutic range.

## Materials and Methods

### Patients and Treatment Regimens

This study was conducted with 128 pediatric patients who received busulfan from July 2018 to February 2021. Patients who received IV busulfan as part of the conditioning regimen for HSCT were included, and patients for whom busulfan PK data were not available due to blood collection difficulties were excluded. This study was approved by the Medical Ethics Committee of the Children’s Hospital of Soochow University (Suzhou, China), and all patients/parents provided written informed consent.

Busulfan (Busulfex; Otsuka Pharmaceutical Co., Ltd.) dosage was 0.8 mg kg^−1^–1.2 mg kg^−1^ based on the actual or adjusted body weight (for obese patients). The adjusted body weight was calculated using the following equation: adjusted body weight = (actual weight − standard weight) × 0.25 + standard weight. The standard weight refers to the standardized growth charts for height and weight of Chinese children and adolescents aged 0–18 years (Hui et al., 2009). The therapeutic dose strata of busulfan are summarized in Supplementary Table S2. Busulfan was administered four times daily over 2 to 4 consecutive days as a 2 h infusion *via* a central venous catheter. No dosage adjustments were performed during the whole therapy procedure.

The pretreatment regimen varied depending on the different underlying diseases and types of donors. Generally, busulfan was combined with either CTX, fludarabine (FLU), cladribine, cytarabine, etoposide or anti-thymocyte globulin. Oral phenytoin was used on the busulfan infusion days as seizure prophylaxis, beginning the day before the initiation of busulfan treatment. Cyclosporine or tacrolimus and mycophenolate mofetil in combination with methotrexate were used for aGVHD prophylaxis. Heparin and alprostadil were used for VOD prophylaxis, and mesna was given to prevent hemorrhagic cystitis.

### Busulfan Pharmacokinetic Analysis and Genotyping

PK blood samples were obtained from the peripheral vein or central venous line (not used to infuse busulfan) at 0, 2, and 4 h (prior to the next scheduled busulfan administration) after the end of the first dose of busulfan infusion. Additionally, samples were collected before the fifth infusion dose for 61 (47.7%) patients. The specific time of sampling was recorded. Two to three milliliters of whole blood were collected in EDTA tubes for each sample. The samples were centrifuged and stored at −80°C until analysis. Busulfan plasma concentrations were measured using optimized liquid chromatography–tandem mass spectrometry (Li-na et al., 2016). The calibration curve was linear over a concentration range of 0.1–10 µg ml^−1^ (r = 0.997), and the lower limit of quantitation was 0.1 µg ml^−1^.

Blood samples for genotyping were withdrawn before the first busulfan infusion. DNA extraction was performed using the QIAamp DNA Blood Mini Kit (Qiagen, Germany), following the manufacturer’s instructions. The genetic variants for *GSTA1* were determined at the following loci: rs3957357, rs3957356, rs11964968, rs4715333, and rs58912740. *GSTP1* was genotyped according to rs1695. Single-nucleotide polymorphisms (SNPs) in the *GSTA1* and *GSTP1* genes were separately genotyped using multiplex PCR and sequencing. A panel containing 6 target SNP sites was designed. Library preparation was performed using two-step PCR. Paired-end sequencing of the library was performed on HiSeq XTen sequencers (Illumina, San Diego, CA).

### Population Pharmacokinetic Modeling

Pop-PK analysis was performed using nonlinear mixed effect model (NONMEM) software (version 7.3.0; Double Precision; ICON Development Solutions). The auxiliary software included Wings for NONMEM (Version 7.4.1, http://wfn.sourceforge.net), R packages (version 3.4.3, http://www.r-project.org) and Pirana (version 2.9.4, http://www.pirana-software.com). The ADVAN1 and TRAN2 subroutines and the first-order conditional estimation method with interaction (FOCE-I) were chosen to estimate parameters.

Busulfan pharmacokinetics were fitted using a one-compartment PK model with first-order elimination as the structural model. The estimated PK parameters included the CL and distribution volume (V). The between-subject (BSV) and between-occasion variability (BOV) were assessed using an exponential model (Supplementary Equation S1). Residual variability was described using a combined model with a proportional and additive model (Supplementary Equation S2).

The variability in CL and V was predicted using body size and composition with a theory-based allometric model and busulfan metabolism maturation upon CL was also evaluated as a maturation function (F*mat*), as proposed by McCune et al. (2014). Nine different models based on normal fat mass (NFM) scaling of CL were assessed using Eq. 1 (Table 1). V was predicted using Eq. 2. The Akaike information criterion (AIC) and Bayesian information criterion (BIC) were estimated for these models using Pirana. Models with lower AIC and BIC values were considered superior models.

$$\text{CL}={\text{CL}}_{\text{STD}}\;\times \;{\left(\frac{\text{NFM}}{{\text{NFM}}_{\text{STD}}}\right)}^{\text{k}1}\times \text{ Fmat}$$ (1)

$$\text{V}={\text{V}}_{\text{STD}}\;\times \;\frac{\text{NFM}}{{\text{NFM}}_{\text{STD}}}$$ (2)

where CL_STD_ and V_STD_ are the typical CL and V values scaled to those of an adult male with a body weight of 70 kg and a height of 176 cm. The standard NFM (NFM_STD_) was calculated using Supplementary Equations S3, S4. The exponent *k*_*1*_ was estimated in seven models, and in Model I and Model III, *k*_*1*_ was fixed to a theory-based value of 3/4 (Holford and Anderson, 2017). F*mat* represents the maturation of CL based on postmenstrual age (PMA) using Supplementary Equations S5, S6 and was fixed to 1, except in Model III.

**TABLE 1:** Parameter estimates of the nine NFM-dependent CL candidate models.

Parameters	Model I	Model II	Model III	Model IV	Model V	Model VI	Model VII	Model VIII	Model IX
	3/4 Allometric model	Simple exponent model	3/4 Allometric and maturation function model	Age-cutoff separated model	Age-cutoff separated model	Weight-dependent exponent model	FFM-dependent exponent model	Age-dependent exponent model	PMA-dependent exponent model
Model description	CL×(NFM/56.1) ^3/4^	CL×(NFM/56.1) ^*k1*^	CL×(NFM/56.1) ^3/4^×F*mat*	CL×(NFM/56.1) ^*k1*^	CL×(NFM/56.1) ^*k1*^	CL×(NFM/56.1) ^*k1*^	CL×(NFM/56.1) ^*k1*^	CL×(NFM/56.1) ^*k1*^	CL×(NFM/56.1) ^*k1*^
OFV	−1305.01	−1306.7	−1307.41	−1308.2	−1308.25	−1313.41	−1311.72	−1310.33	−1310.09
AIC	−1291.01	−1290.7	−1289.41	−1288.2	−1288.25	−1291.41	−1289.72	−1288.33	−1288.09
BIC	−1261.98	−1257.53	−1252.1	−1246.73	−1246.78	−1245.8	−1244.11	−1242.72	−1242.48
CL_STD (L h^−1^)	7.5	7.98	7.71	21.3^a^/7.86^b^	11.7^c^/7.86^d^	7.79	7.87	7.73	7.74
V_STD (L)	42.4	42.4	42.4	42.4	42.4	42.4	42.4	42.4	42.4
RUV_PROP	0.131	0.131	0.13	0.13	0.131	0.13	0.13	0.13	0.13
RUV_ADD	0.0479	0.0479	0.048	0.0479	0.0477	0.0478	0.0477	0.0479	0.0479
F*fat*_CL	0.654	0.625	0.692	0.66	0.683	0.771	0.748	0.725	0.72
*k*_*1*_	0.75	0.801	0.75	1.31^a^/0.783^b^	1.0^c^/0.785^d^		-	-	-
*k*_*1*_ = *k*_*0*_–*kmax*/{[1+ (Weight or FFM/*k*_*50*_)^−HILL^]} or *k*_*1*_ = *k*_*0*_–k*max*/{[1+ (Age or PMA/*k*_*50*_)^−HILL^]}									
TM_50_	-	-	31	-	-	-	-	-	-
*k*_*0*_	-	-	-	-	-	0.938	0.985	0.838	0.832
*kmax*	-	-	-	-	-	0.169	0.205	0.0808	0.0731
*k*_*50*_	-	-	-	-	-	8.46	5.85	1.4	115
HILL	-	-	2.03	-	-	964	365	285	1080
BSV_CL	0.237	0.235	0.234	0.234	0.233	0.229	0.23	0.231	0.231
BSV_V	0.24	0.24	0.24	0.241	0.24	0.241	0.241	0.241	0.241

a Estimates for children ≤1 year of age.

b Estimates for children >1 year of age.

c Estimates for children ≤2 years of age.

d Estimates for children >2 years of age.

OFV, objective function value; AIC, akaike information criterion; BIC, bayesian information criterion; Clearance (CL_STD) and distribution volume (V_STD) estimates are standardized for an adult male with a bodyweight of 70 kg and a height of 176 cm (FFM_STD_ = 56.1 kg); RUV_PROP, proportional residual unidentified variability; RUV_ADD, additive residual unidentified variability; F*fat*_*CL*, fat fraction for CL; TM_50_, postmenstrual age (PMA) when busulfan metabolism reaches 50% of adult levels; *kmax* is the maximum decrease of the exponent, *k*_*50*_ is the weight (Model VI and VII) or age (Model VIII and IX) at which a 50% decrease in the maximum decrease is attained; HILL, hill coefficient for maturation; BSV, between-subject variability, implicated in CL and V.

Other potential covariates included the baseline disease (malignant vs. nonmalignant), hematological and biological indicators, genotype and daily dosage of coadministered drugs (including fludarabine, phenytoin and metronidazole), which were collected from the patients’ medical records. The continuous covariates were introduced into the model as Supplementary Equation S7. The categorical covariates were introduced into the model as Supplementary Equation S8. All covariates were introduced into the basic model individually to identify the covariates with statistical significance for the busulfan PK parameters. The significance level was set to 0.05 (*df* = 1, change in objective function value (OFV) = 3.84). The analysis of potential covariates was further performed using a stepwise procedure based on the changes in OFV. During forward selection, the significance level was set to 0.01 (*df* = 1, change in OFV = 6.64). During backward elimination, the significance level was set to 0.001 (*df* = 1, change in OFV = 10.83).

The accuracy and robustness of the final model were evaluated using bootstrap methods, goodness-of-fit plots and prediction-corrected visual predictive checking (pc-VPC). Once the final Pop-PK model was established, it was used to provide estimates of individual busulfan AUC for subsequent evaluations of the relationships with transplantation outcomes.

### Correlation Analysis Between Busulfan Area Under the Concentration–Time Curve and Clinical Outcomes

The main study endpoint was event-free survival (EFS), as calculated from the time of transplant until graft failure, relapse of disease, or death, whichever occurred first (Philippe et al., 2016). First, the busulfan AUC was taken as a continuous variable, with a quadratic function to describe the correlation between log (AUC) and the log hazard of an event. The busulfan AUC range corresponding to a negative log hazard of an event was considered the optimum range. Second, the busulfan AUC was evaluated as a categorical variable (within or outside of the optimum range), together with age, weight, sex, disease type, donor source, and HLA disparity, using univariate and multivariate Cox proportional hazards regression models.

The secondary study endpoint was TRTs, including VOD, grade II–IV aGVHD, mucositis and hemorrhagic cystitis. VOD was diagnosed according to the modified Seattle criteria (Dignan et al., 2013), aGVHD was diagnosed and graded according to the Mount Sinai Acute GVHD International Consortium (MAGIC) criteria (Harris et al., 2016), and oral mucositis was evaluated and scored based on the ESMO Clinical Practice Guidelines (Peterson et al., 2015). The cumulative incidence of TRTs was evaluated with graft failure, relapse, and death as competing events. The correlation of TRTs incidence with the busulfan AUC was analyzed using logistic regression. All computations were conducted in R (version 4.1.0) with the *survival*, *survminer*, and *ezcox* R packages. *p <* 0.05 was regarded as significant.

## Results

### Patient Characteristics

A total of 467 blood samples obtained from 128 enrolled patients were collected for Pop-PK model development. The patient characteristics and clinical laboratory results for the patients are summarized in Supplementary Table S3. Because samples for genotyping were not available from two patients, the *GSTA1* and *GSTP1* genotyping results were obtained for 126 patients and are summarized in Supplementary Table S4. Both the G*STA1* and *GSTP1* genetic frequencies for the patients were in Hardy–Weinberg equilibrium.

### Population Pharmacokinetic Model Development

Among the nine various NFM-dependent CL models examined (Table 1), although the AIC value for the weight-dependent exponential model (Model VI) was the smallest, the larger HILL index value resulted in *k*_*1*_ being approximately equal to 0.769, which was close to the fixed value for the allometric exponent of 3/4 in Model III. Meanwhile, the F*mat* parameter introduced in Model III could better predict the effect of body size, physiological function, and body maturity on CL. Therefore, Model III was finally chosen as the basic structural model.

In the initial covariates screening process, the disease status (malignant or nonmalignant), *GSTA1,* and *GSTP1* gene polymorphisms had no statistical significance on the PK parameters of busulfan. During forward selection, the results showed that urea nitrogen (UREA) and γ-glutamyl transpeptidase (GGT) had statistical significance, but the correlation coefficient was 0.244 for UREA on CL and −0.1 for GGT on V, which was inconsistent with physiological function. Therefore, these two covariates were eliminated from the model, and then the final model was established. The potential covariate screening results are shown in Supplementary Table S5.

The parameter estimates of the final model and bootstrap are summarized in Table 2. The final model indicated that the typical population estimates of CL and V were 7.71 L h^−1^ and 42.4 L, respectively. The value of the fraction of fat mass estimated for CL (F*fat*_CL) was 0.692, and that for V was zero. These values indicated that NFM and F*mat* were suitable covariates for explaining variability in CL, while FFM was the covariate for explaining variability in V. The BSV values for CL and V were 23.4% and 24.0%, respectively.

**TABLE 2:** Final Pop-PK model for busulfan: PK parameter estimates and bootstrap results.

Parameter	Original	Average	95% CIs		RSE (%)	Shrinkage (%)
CL_STD (L h^−1^)	7.71	7.90	7.30	9.44	10.6	
V_STD (L)	42.4	42.4	40.4	44.5	2.6	
RUV_PROP	0.130	0.130	0.092	0.164	13.7	
RUV_ADD (mg L^−1^)	0.048	0.046	0.016	0.065	26.2	
F*fat*_CL	0.692	0.702	0.196	1.301	38.7	
TM_50_ (weeks)	31.0	35.2	8.2	79.5	58.1	
HILL	2.03	2.97	0.44	9.92	81.1	
BSV_CL	0.234	0.229	0.195	0.265	7.9	8.24
BSV_V	0.240	0.238	0.191	0.287	10.1	15.9

CI, confidence interval; RSE, relative standard error; Clearance (CL_STD) and distribution volume (V_STD) estimates are standardized for an adult male with a bodyweight of 70 kg and a height of 176 cm (FFM_STD_ = 56.1 kg); RUV_PROP, proportional residual unidentified variability; RUV_ADD, additive residual unidentified variability; F*fat*_CL, fat fraction for CL; TM_50_, postmenstrual age when busulfan metabolism reaches 50% of adult levels; HILL, hill coefficient for maturation; BSV, between-subject variability, implicated in CL and V.

**FIGURE 1:**
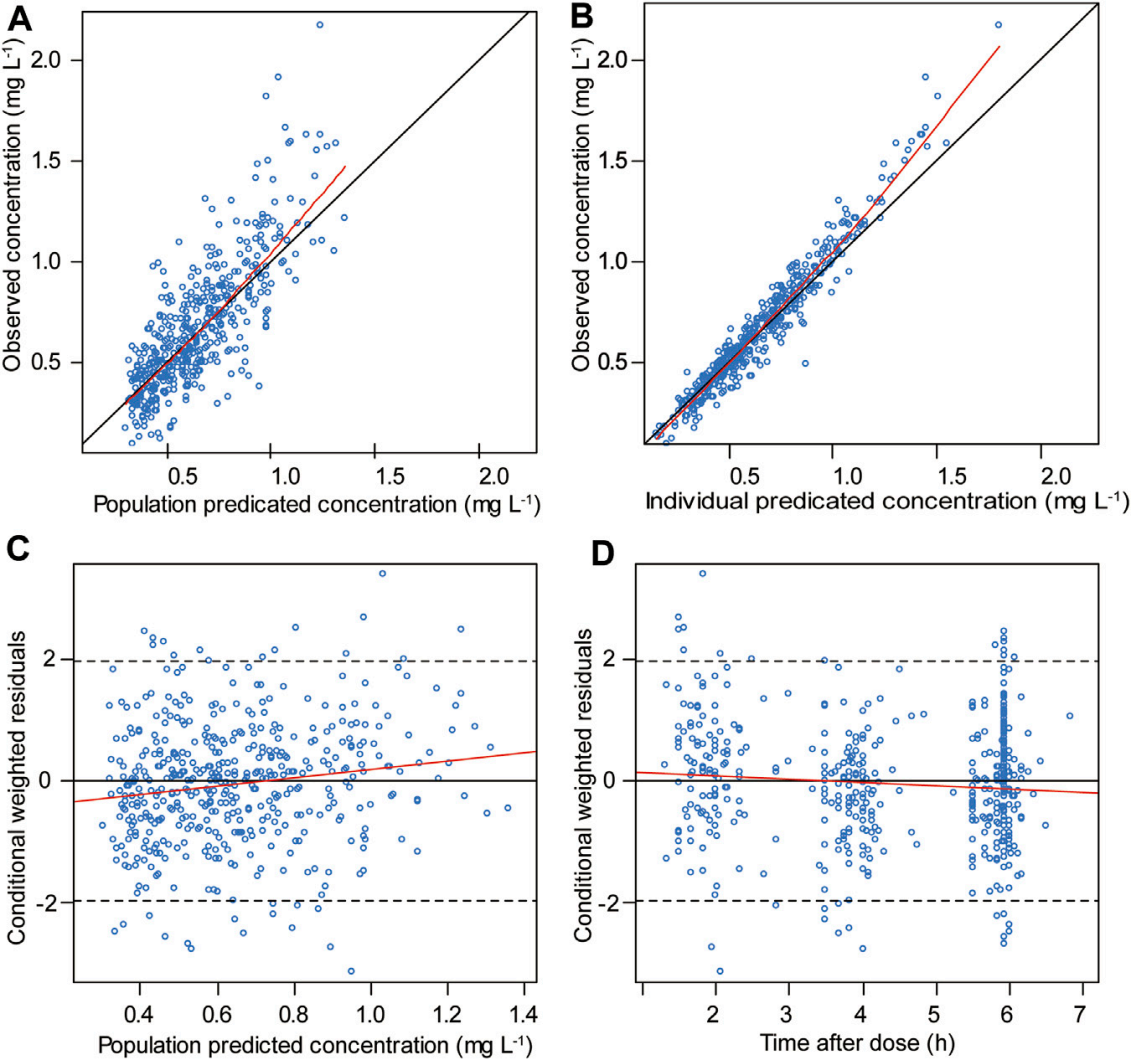
Goodness-of-fit plots for the final model included the following: **(A)** observed vs. population predicted concentrations, **(B)** observed vs. individual predicted concentrations, **(C)** conditional weighted residuals vs. population predicted concentrations and **(D)** conditional weighted residuals vs. time after the first dose. The red line represents the fitted line.

**FIGURE 2:**
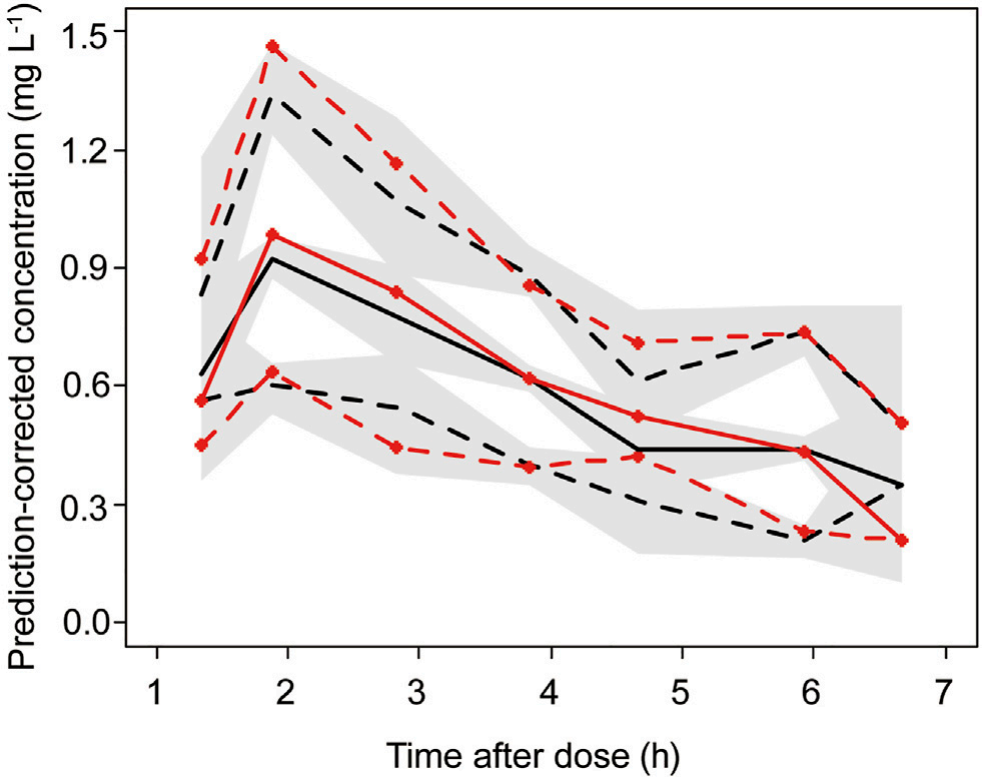
Final model prediction-corrected visual prediction check (pc-VPC) of busulfan concentration. Observed (with symbols) and predicted 5th, 50th and 95th percentiles, with predicted 95% CIs (shaded regions). The red line represents the observed concentrations, and the black line represents the predicted concentrations.

The goodness-of-fit plots for the final model are shown in Figure 1. The pc-VPC plot for the final model is shown in Figure 2.

### Correlations Between the Busulfan Area Under the Concentration–Time Curve and Clinical Outcomes

To avoid the variation in busulfan exposure caused by different busulfan administration days, this part of the study only included patients received 16-dose busulfan myeloablative regimen (4 times daily over 4 days). The demographic and transplantation characteristics of the 63 patients are summarized in Table 3. The median busulfan AUC obtained from the Pop-PK model was 1,425.4 µM × min (range 691.5–2156.0 µM × min). With a median follow-up of 24.8 months (6.8–32.8 months), a total of 16 events occurred, including graft failure in 2 patients, disease relapse in 8 patients (four patients died) and 7 patient deaths (one with graft failure and six not related to relapse). The event summary information is listed in Supplementary Table S6.

**TABLE 3:** Characteristics of patients treated with the 16-dose busulfan regimen (*n* = 63).

Demographic characteristics	Median (range)/number (%)
Follow-up (months)	24.8 (6.8–32.8)
Age (years)	2.5 (0.6–16.9)
Bodyweight (kg)	14.4 (7–63)
Busulfan AUC (μM × min)	1425.4 (691.5–2156.0)
Sex	
Male	44 (69.8%)
Female	19 (30.2%)
Diagnosis	
Malignant	50 (79.4%)
Acute myeloid leukemia (AML)	24 (38.1%)
Acute lymphoblastic leukemia (ALL)	22 (34.9%)
Myelodysplastic (MDS)	1 (1.6%)
Myeloproliferative neoplasms (MPN)	1 (1.6%)
Juvenile myelomonocytic leukemia (JMML)	2 (3.2%)
Nonmalignant	13 (20.6%)
Wiskott-Aldrich syndrome (WAS)	8 (12.7%)
Thalassemia	4 (6.3%)
Severe aplastic anemia (SAA)	1 (1.6%)
HLA disparity	
Matched	5 (7.9%)
Mismatched	58 (92.1%)
Cell source	
Umbilical cord blood	31 (49.2%)
Peripheral blood stem cell	12 (19.0%)
Bone marrow or peripheral blood stem cell and bone marrow combined	20 (31.7%)
Events	
Graft failure	2 (3.2%)
Disease relapse	8 (17.4%)
Death	11 (17.5%)

**FIGURE 3:**
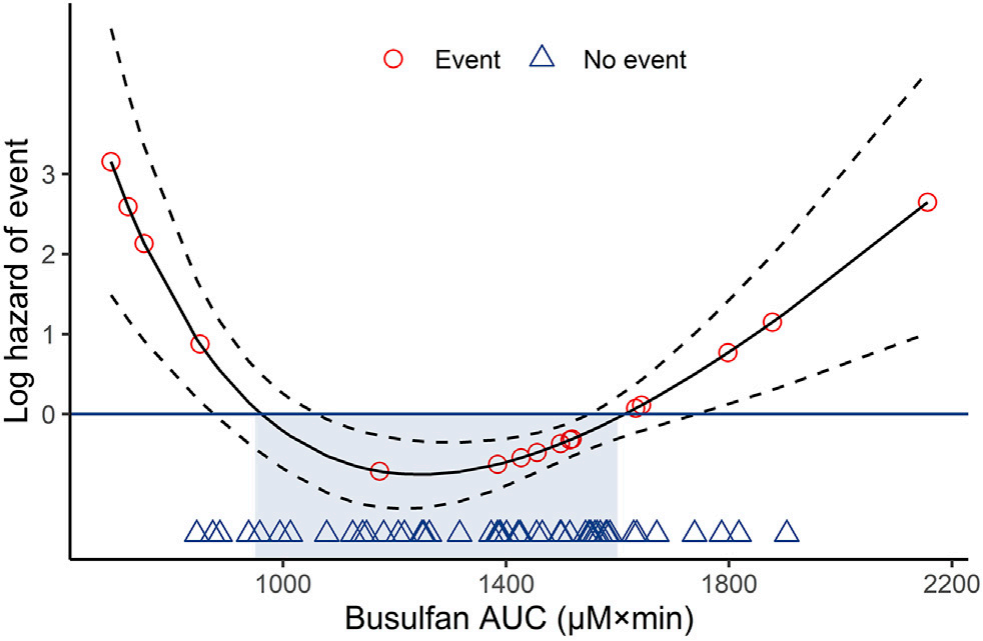
Log hazard risk model for event-free survival calculated as a quadratic function. Dotted lines represent the 95% confidence intervals. AUC, area under the curve.

After HSCT, the estimated EFS was 74.5% (95% CI: 64.4–86.1) at 2 years. In the log hazard model, the estimated hazard of events as a function of busulfan AUC suggested the existence of an optimal interval. The model produced an optimum busulfan AUC of 950–1,600 µM × min, as shown in Figure 3.

**FIGURE 4:**
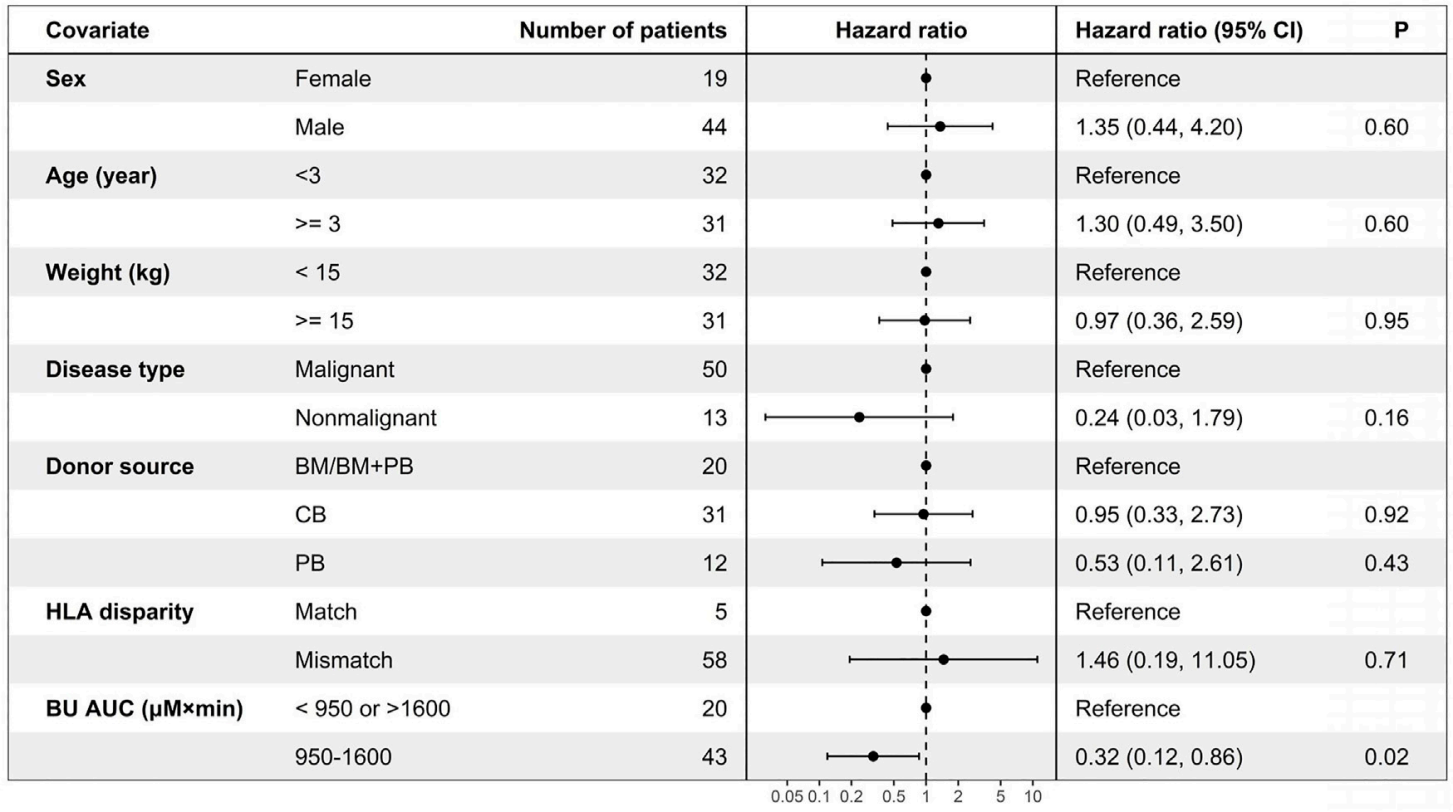
Forest plot of univariate Cox regression analysis for event-free survival (*n* = 63).

**FIGURE 5:**
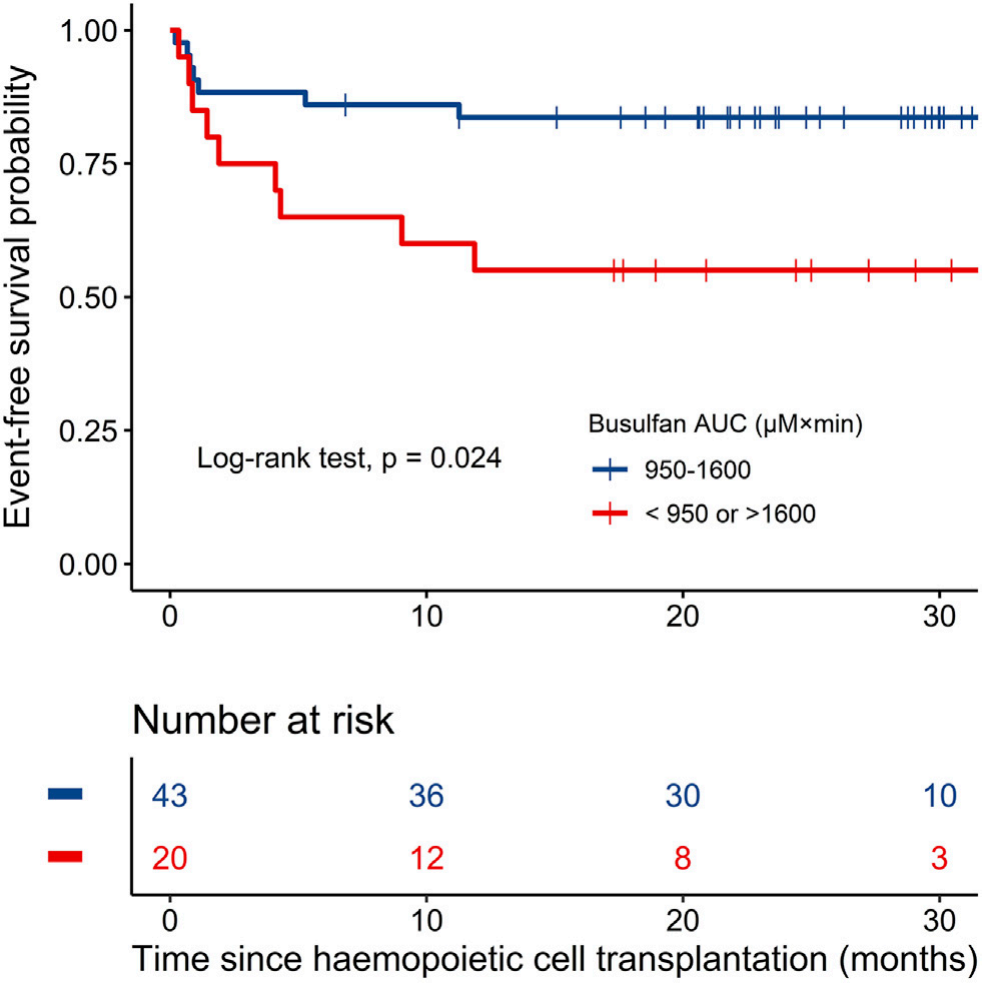
Kaplan–Meier plots of event-free survival in the two groups based on busulfan AUC (*n* = 63).

In the univariate and multivariate Cox regression analyses, the busulfan AUC was identified as the only significant factor for EFS (hazard ratio (HR) 0.32, 95% CI: 0.12–0.86; *p* = 0.02), as shown by the forest map in Figure 4, while weight, sex, disease type, donor source, and HLA disparity did not affect EFS. Kaplan–Meier curves were used to compare the EFS of patients inside the optimum AUC interval with that of patients outside the interval. Patients with an AUC of 950–1,600 µM × min had 83.7% (95% CI: 73.3–95.5) EFS compared with 55.0% (95% CI: 37.0–81.8) for patients with low (<950 µM × min) or high (>1,600 µM × min) exposure (*p* = 0.024; Figure 5).

Grade II-IV aGVHD developed in 17 (27.0%) patients (grade II in 4 patients, grade III in 6 patients, and grade IV in 7 patients), with a cumulative incidence of 26.98% (95% CI: 15.9–38.1) at 100 days. In the absence of VOD, hepatotoxicity (serum bilirubin) of at least grade 2 until 30 days after transplantation was evaluated and graded according to the Common Terminology Criteria for Adverse Events (v5.0). Hepatotoxicity occurred in 18 patients (grade II in 17 patients and grade III in one patient). The cumulative incidences of hepatotoxicity, oral mucositis (in 10 patients) and hemorrhagic cystitis (in 3 patients) at 30 days posttransplantation were 28.6% (95% CI: 17.3–39.8), 15.9% (95% CI: 6.7–25.0), and 4.8% (95% CI: 0.00–10.1), respectively. The logistic regression analysis results showed no association between these toxicity complications and the busulfan AUC (*p* > 0.05, Supplementary Figure S1).

## Discussion

This study used Pop-PK analysis to characterize the relationships between busulfan exposure and clinical outcomes in Chinese pediatric patients with HSCT. With the developed Pop-PK model and using physiologically-based descriptions of body composition and theory-based allometric principles, CL for busulfan was estimated according to allometry NFM and F*mat*, and V was estimated according to FFM. Furthermore, the results demonstrated that an AUC of 950–1,600 µM × min was associated with better EFS in children receiving the 16-dose busulfan regimen before HSCT.

The typical population estimates for busulfan CL and V standardized for an adult patient weighing 70 kg were 7.71 L h^−1^ and 42.4 L in the final model, respectively. The estimated V was in good accordance with other reports (McCune et al., 2014; Rhee et al., 2017; Nava et al., 2018; Yuan et al., 2021). The estimated CL was slightly lower than that in prior studies from Korea (10.7 L h^−1^) (Rhee et al., 2017), Caucasian (11.4 and 13.58 L h^−1^) (McCune et al., 2014; Nava et al., 2018) and Chinese (11.08 L h^−1^) (Yuan et al., 2021) pediatric patients. However, in a recently published study (Dunn et al., 2021), the typical CL for a 20 kg child was 1.14 L h^−1^, which were reduced estimates compared to ours and prior studies. These discrepancies in CL might be due to published models contain various structural PK models, various covariates, different age distributions and ethnicities (Huang et al., 2022).

It is well-known that body weight, age and body surface area (BSA) have significant impacts on busulfan PK in pediatric patients (Nguyen et al., 2004; Booth et al., 2007; Trame et al., 2011; Bartelink et al., 2012; Savic et al., 2013; McCune et al., 2014; Wang et al., 2015; Rhee et al., 2017). NFM, a theory-based size descriptor that divides body weight into FFM and fat mass, was used to estimate the effect of body size and composition on busulfan PK in infants to adults (McCune et al., 2014; van Hoogdalem et al., 2020). In the present analysis, nine models were proposed to characterize the PK of busulfan by introducing the NFM parameter. In the final model, 69.2% fat mass in addition to FFM described CL for busulfan, whereas the F*fat* was zero for V. As proposed by McCune et al., the fraction of the fat mass was 50.9% for CL and 20.3% for V (McCune et al., 2014). The different fractions of fat mass may be caused by the different age distributions in the different study groups. In McCune et al. (2014) study, infant and adult patients were included, with a wide age range (0.1–65.8 years). All patients included in the present study were children, with an average age of 6.1 years (0.6–17 years). The maturation of CL is more appropriately described by PMA than by postnatal age because the maturation of CL begins before birth (Anderson and Holford, 2009). The PMA was calculated by adding postnatal age to gestational age. In the current study, the maturation of the busulfan CL reached 50% of adult values at 31 weeks of PMA, considering body composition, which was lower than in another study (TM_50_ = 45.7) (McCune et al., 2014). In our cohort, the actual gestational ages of the patients were recorded, whereas, in McCune et al. (2014) study, the values were assumed to be 40 weeks due to missing information. This might be the reason for the bias. In two recent Pop-PK studies of busulfan based on Chinese pediatric patients, BSA was considered one of the significant covariates for CL in the model developed by Yuan et al. (2021). Huang et al. (2022) conducted a systemic external evaluation of 11 published models of busulfan based on an independent dataset from 40 Chinese pediatric populations and similarly concluded that a model developed from the Korean population with BSA affecting CL as one of the covariates had the most satisfactory predictability for Chinese children (Huang et al., 2022). Based on children’s growth and developmental characteristics, CL in the pediatric population should be investigated using models that account for the influences of body size, maturation, and organ function. Allometric scaling using an empiric power exponent of 3/4 is superior to scaling using BSA, which has already been described by Anderson and Holford (Anderson and Holford, 2009).

After including body composition and maturation in the model, several other potential covariates (including disease type, concomitant medications, hematological and biological indicators, and genetic variants of *GSTA1* or *GSTP1*) had no significant effect on busulfan PK. The guideline from the American Society for Blood and Bone Marrow Transplantation (ASBMT) stated that fludarabine, deferasirox, and metronidazole have an impact on IV busulfan CL. The effect of phenytoin on IV busulfan CL is unclear, although it is known to affect oral busulfan CL (Palmer et al., 2016). However, none of these three drugs were found to have an effect on IV busulfan PK in the present study. This finding was consistent with the results of a recent report, which showed that children with HSCT did not experience significant effects on busulfan CL when coadministered with drugs that could theoretically interfere with busulfan metabolism (Dunn et al., 2021). Busulfan is mainly metabolized and eliminated by the liver. Elevated alanine transaminase (ALT) and aspartate transaminase (AST) levels are indicative of potential liver damage, thus affecting the elimination of busulfan. Two cohorts of Asian pediatric patients showed that increased AST levels affected busulfan CL (Rhee et al., 2017; Yuan et al., 2021). However, ALT or AST enzymes were not found to be significant covariates with busulfan CL in the present study, which is in line with another report on pediatric Fanconi anemia patients (van Hoogdalem et al., 2020). This might be explained by the fact that only a few cases (7 cases) of our children had both abnormally high ALT and AST levels. In the study by Bartelink et al. (2012), it was also shown that neither biochemical parameters nor blood counts could predict individual variability (Bartelink et al., 2012). The influence of GST gene polymorphisms on busulfan CL remains controversial (Kim et al., 2019). However, the present study and other modeling studies failed to incorporate the genetic variants of *GSTA1* and *GSTP1* as covariates into the final model (Zwaveling et al., 2008; Sun et al., 2020). Models established in pediatric populations could suggest that a validated pharmacogenetics-based Pop-PK model would be a beneficial tool for individualized busulfan administration (Nava et al., 2017; Nava et al., 2018; Yuan et al., 2021).

The AUC 900–1,350 µM × min or 900–1,500 µM × min has been widely used as a target busulfan exposure to improve clinical outcomes in children with HSCT (Bolinger et al., 2001; Ansari et al., 2014; Philippe et al., 2016). A recent report showed that Chinese children with a target exposure of 900–1,350 µM × min also had a better survival outcome (Shao et al., 2022). In the present study of 63 children on a 16-dose busulfan pretreatment regimen, we found that patients with an AUC of 950–1,600 µM × min achieved optimal EFS, which is slightly higher than the target exposure recommended by the FDA or EMA dosage regimens for children. A previous retrospective study conducted by Bartelink et al. (2009) suggested that a total AUC of 74–82 mg h L^−1^ (∼AUC 1125–1,250 µM × min per dose) was associated with the highest EFS in children with malignant and nonmalignant diseases (Bartelink et al., 2009). A multicenter study from 15 different transplantation centers defined that the optimum cumulative AUCs of 78–101 mg h L^−1^ (∼AUC 1225–1,575 µM × min per dose) predicted higher EFS in children and young adults than those in lower- and higher-exposure groups (Bartelink et al., 2016). This upper threshold of 1,575 µM × min is generally consistent with 1,600 µM × min in this study, showing that this threshold is safe and associated with low toxicity. Notably, Ansari et al. indicated that Css >600 ng ml^−1^ (∼AUC >900 µM × min) was independently associated with lower EFS by multivariate analysis (Ansari et al., 2014). Benadiba et al. (2018) also observed reduced EFS in patients with Css >600 ng ml^−1^ compared with patients with Css <600 ng ml^−1^ in children with myeloid malignancies receiving unrelated umbilical cord blood transplantation (Benadiba et al., 2018). The differences in the optimal exposure of busulfan between these studies may be influenced by multiple factors, including age, donor source, disease diagnosis, and other myeloablative agents included in the preparative regimen (Bartelink et al., 2016; Shukla et al., 2020). Despite the bias, these findings suggest that busulfan exposure is strongly associated with clinical outcomes in children and that establishing an optimal target exposure is warranted.

No significant correlations between busulfan AUC and TRTs were identified in the current analysis. This is consistent with the findings reported in a recent study in a Chinese pediatric population. This literature showed that busulfan exposure could not be used to predict VOD, liver injury, grade II-IV aGVHD, hemorrhagic cystitis or oral mucositis after multivariate analyses (Shao et al., 2022). As early as 30 years ago, the busulfan AUC of 1,500 µM × min was considered the upper threshold because higher exposure was associated with an increased risk of VOD in adults (Grochow et al., 1989). For safety reasons, this threshold was also used in pediatric patients, although this correlation remains controversial according to previous studies using IV busulfan in children (Bartelink et al., 2009; Malär et al., 2011; Ansari et al., 2014; Philippe et al., 2016). Since all patients in the current cohort did not have VOD, the association between high busulfan exposure and VOD could not be established. A previous study done by Philippe et al. reported that all children who developed VOD had a busulfan AUC of <1,500 µM × min (Philippe et al., 2016). In another large cohort, also by Philippe et al. (2019), which specifically looked at the determinants of VOD, an increased risk of VOD was observed in children with a maximum busulfan concentration (Cmax) value of ≥1.88 ng ml^−1^ (Philippe et al., 2019). In addition to the high busulfan exposure, several other factors, such as underlying disease, genetic polymorphisms, iron overload, hepatic fibrosis or cirrhosis, influence the incidence of VOD (Dignan et al., 2013; Philippe et al., 2019). At present, the correlation between aGVHD and busulfan exposure is not clear, and conflicting results exist. Some studies have shown that higher busulfan exposure was associated with a higher incidence of grade II-IV aGVHD (Bartelink et al., 2009; Ansari et al., 2014); however, other researchers have found that the risk of aGVHD increased with low exposure (Russell et al., 2013). Similar to our study, some previous investigations found no association between busulfan exposure and grade II-IV aGVHD in children (Baker et al., 2000; Shao et al., 2022). Although a previous study showed a positive correlation between busulfan AUC and the severity of stomatitis in children (Michel et al., 2012), the correlation was not found in this study, which was also consistent with other studies (Bartelink et al., 2009; Ansari et al., 2014). Several studies have emphasized that the combined use of melphalan may increase the risk of stomatitis because melphalan is metabolized by the same enzyme system as busulfan (Bartelink et al., 2009; Michel et al., 2012). Although one study showed that the cumulative incidence of hemorrhagic cystitis was higher for patients with a Css >600 ng ml^−1^ compared to patients with Css <600 ng ml^−1^ (Benadiba et al., 2018), this correlation was not observed in our study and another study (Ansari et al., 2014). These differences may be explained by different populations and conditioning regimens. Other factors must be further studied to identify patients at risk for such toxicities.

However, the present study has several limitations. It included a limited number of patients from a single center. The predictive performance of the final Pop-PK model has yet to undergo external validation. Moreover, several other genetic covariates for busulfan PK have not been assessed in the present analysis, such as the *GSTM1*-null and CYP39A1 genotypes, which were found in association with busulfan PK (Ten Brink et al., 2013; Ansari et al., 2017). In addition, other factors might have affected clinical outcomes, such as the disease grading and remission status of malignant disease before transplantation, GVHD prophylaxis regimens before and after transplantation could not be included in the present analysis (Bartelink et al., 2016). A multicenter trial is ongoing (ClinicalTrials.gov: NCT04786002), aiming to establish the optimal busulfan treatment window for myeloablative conditioning in Chinese pediatric patients and confirm our preliminary results.

## Conclusion

In conclusion, the final Pop-PK model established in the current study shows that allometric FFM, F*fat* and F*mat* can describe the variability in busulfan CL in pediatric patients and that FFM can be used to describe the variability in V, while fat mass has no correlation with V. The result of the present study suggests that busulfan exposure (an AUC of 950–1,600 µM × min) was associated with better EFS in children receiving a 16-dose busulfan myeloablative conditioning regimen before HSCT. The performance of the present Pop-PK model and the preliminarily established target busulfan exposure need to be further validated in prospective studies with larger samples.

## Data Availability

The original contributions presented in the study are included in the article/Supplementary Material, further inquiries can be directed to the corresponding authors.

## Supplementary Material

Supplementary Figure S1 Logistic regression for the correlation of transplant-related toxicities with the busulfan AUC.
