# Supplementary figures and images for: The Correlation Between Busulfan Exposure and Clinical Outcomes in Chinese Pediatric Patients: A Population Pharmacokinetic Study
Source: Front Pharmacol. 2022 Jun 16;13:905879. doi: 10.3389/fphar.2022.905879 (PMC9243314; doi:10.3389/fphar.2022.905879)

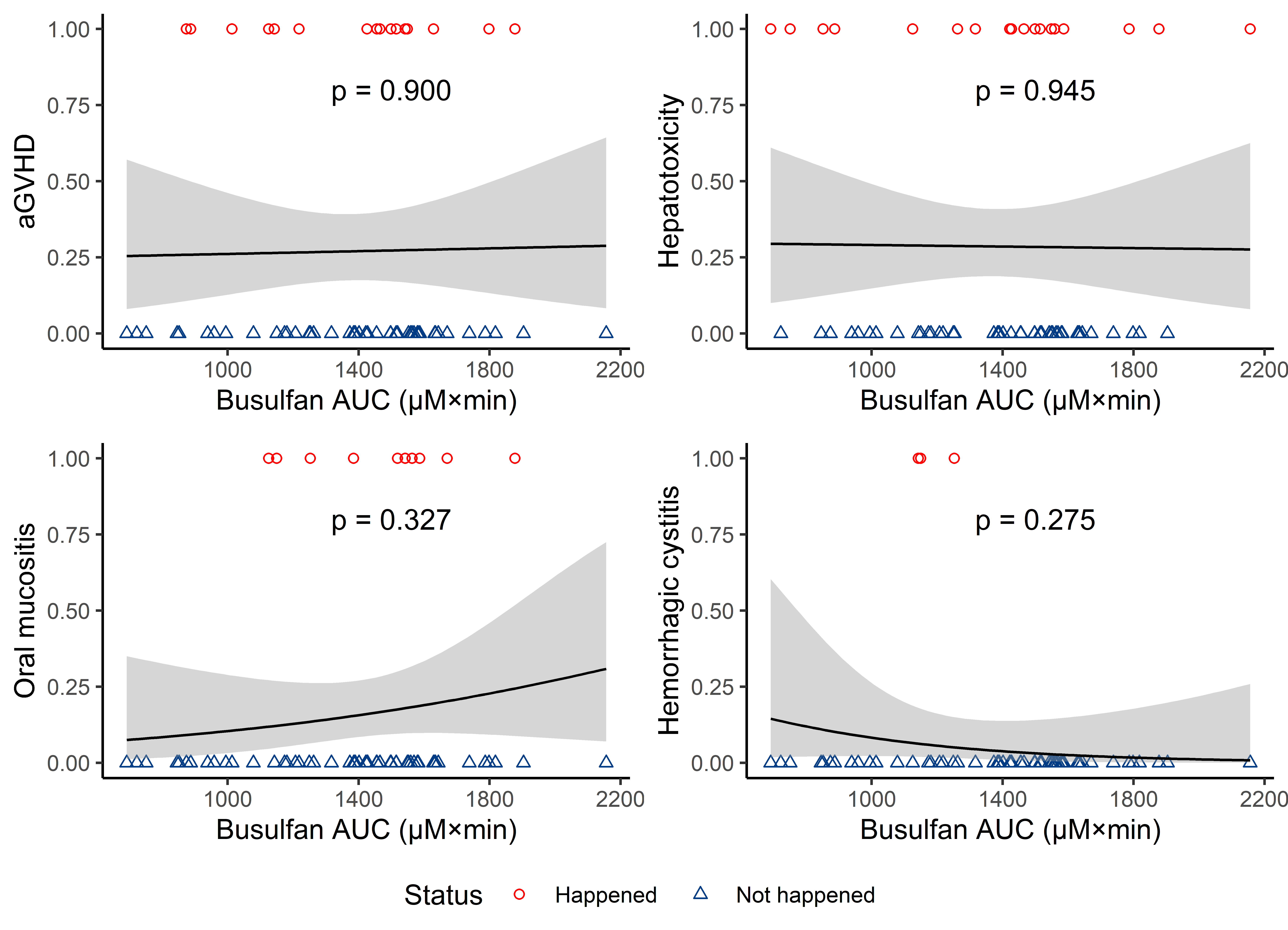

Supplement: Supplementary file 1 [file Image1.JPEG]
