# Supplementary material for: The Correlation Between Busulfan Exposure and Clinical Outcomes in Chinese Pediatric Patients: A Population Pharmacokinetic Study
Source: Front Pharmacol. 2022 Jun 16;13:905879. doi: 10.3389/fphar.2022.905879 (PMC9243314; doi:10.3389/fphar.2022.905879)
Supplement: Supplementary file 2 [file DataSheet1.docx]

Supplementary Material

# Table S1. Between subject and between-occasion variability and residual variability models and equations used in modelling

| **Between subject variability (BSV) and between-occasion variability (BOV) model:**  *P_ij_* represents the i-th individual value of the parameter on the j-th occasion, *TVP_j_* is the typical population value of the parameter, η_ij_ is the between subject variability of the PK parameter, and is normally distributed with a mean of 0 and a variance of ω2. κ represents the between occasion variability. κ was normally distributed with a mean of 0 and a variance of π2. | $P_{ij}={TVP}_{j}\times e^{\eta ij+\kappa_{ij}}$ | (S1) |
| --- | --- | --- |
| **The residual variability model:**  Y represents the observed values, *Con* is the individual predicted busulfan concentration, θ_PROP_ represents the parameter of the proportion residual error, θ_ADD_ represents the parameter of additional residual error, and ε represents the residual error and is assumed to be normally distributed with a mean of 0 and a variance of σ2. | $Y=Con+sqrt\left( {Con}^{2} {\theta_{PROP}}^{2}+ {\theta_{ADD}}^{2} \right) \varepsilon$ | (S2) |
| **FFM (free fat mass, kg):**  WHS*max* is the maximum FFM for any given height (HT, m) and WHS_50_ is the ABW value when FFM reaches 50% of WHS*max*. For males, WHS*max* is 42.92 kg/m^2^ and WHS_50_ is 30.93 kg/m^2^; for females, WHS*max* is 37.99 kg/m^2^ and WHS_50_ is 35.98 kg/m^2^. | $FFM={WHS}_{max} {HT}^{2} \frac{ABW}{{WHS}_{50} {HT}^{2}+ABW}$ | (S3) |
| **NFM (normal fat mass, kg):**  The fraction of fat mass (F*_fat_*) represents the fat mass (ABW-FFM) equivalent to FFM in terms of allometric size. | ${NFM}_{i}={FFM}_{i}+Ffat ({ABW}_{i}-{FFM}_{i})$ | (S4) |
| F*mat* is the fraction of the adult busulfan clearance value; TM_50_ (weeks) is the value of PMA when maturation reaches 50% of the adult clearance; *Hill* defines the steepness of the change with PMA (Anderson and Holford, 2009). | $F_{mat}=\left( \frac{1}{1+\left( \frac{PMA}{TM50} \right)^{-Hill}} \right)$ | (S5) |
| PMA (post-menstrual age, weeks); PW (weeks), gestational age | $PMA=\left( Age\times52 \right)+$*PW* | (S6) |
| θ is the typical value of the population. θ_1_ is the average of the population. θ_2_ is the estimated coefficient of the covariate. | $\theta=\theta_{1}\times{(\frac{Covariate}{mean})}^{\theta_{2}}$ | (S7) |
|  | $\theta=\theta_{1}\times(1+\theta_{2}\times Covariate)$ | (S8) |

# Table S2. Therapeutic dose strata of intravenous busulfan

| **Busulfan dose regimen, mg kg^-1^** | **0.8** | **1** | **1.2** |
| --- | --- | --- | --- |
| Number of patients | 112 | 13 | 3 |
| Actual body weight of patients, kg (mean ± SD) | 25.38 ± 16.55 | 14.65 ± 6.31 | 12.67 ± 1.89 |
| Busulfan single dose, mg (mean ± SD) | 19.58 ± 11.81 | 14.64 ± 6.36 | 15.30 ± 2.95 |

**Table S3. Demographic characteristics and clinical laboratory results for Pop-PK modelling**

| **Demographic characteristics** | **Number of patients in each category (%) or mean ± SD (range)** |
| --- | --- |
| Gender, male/female | 90 (70%) /38 (30%) |
| Diagnosis, Malignant /Non-malignant | 89 (70%) /39 (30%) |
| Post-natal age (years) | 6.11±4.31 (0.6-17.0) |
| Gestational age (weeks) | 39.82±0.94 (32-40) |
| Post-menstrual age (weeks) | 368.32±222.06 (70.6-926) |
| Height (cm) | 115.11±30.55 (67-185) |
| Actual body weight (kg) | 23.99±16.03 (7.5-96.5) |
| Co-administered drugs doses (mg) ^a^ |  |
| Fludarabine | 26.86±11.11 (11.1-67.0) |
| Phenytoin | 202.59±117.14 (60-654) |
| Metronidazole | 636.37±411.89 (100-1800) |
| Laboratory data |  |
| Prealbumin (PA, mg/L) | 211.34±51.45 (86-439) |
| Total protein (TP, g/L) | 59.68±5.84 (33.8-77.8) |
| Albumin (ALB, g/L) | 40.29±3.40 (27.1-50.8) |
| Globulin (GLB, g/L) | 19.39±4.12 (10.5-33.4) |
| Alanine transaminase (ALT, U/L) | 30.56±43.60 (1.4-577.2) |
| Aspartate transaminase (AST, U/L) | 30.14±26.62 (7.9-202.1) |
| Alkaline phosphatase (ALP, U/L) | 171.72±71.78 (52-580) |
| γ-glutamyl transpeptidase (GGT, U/L) | 32.83±44.63 (5.3-390.6) |
| Cholinesterase (CHE, U/L) | 7217.52±1864.21 (2275-13060) |
| Total bilirubin (TBIL, µmol/L) | 9.61±6.56 (1.2-52.5) |
| Direct bilirubin (DBIL, µmol/L) | 3.62±2.10 (0.79-12.12) |
| Indirect bilirubin (IBIL, µmol/L) | 5.99±4.88 (0.41-43.04) |
| Urea nitrogen (UREA, mmol/L) | 3.22±1.31 (0.96-12.79) |
| Serum creatinine (CrS, µmol/L) | 26.46±13.18 (6.7-98.5) |
| Uric acid (UA, µmol/L) | 249.43±86.36 (93.9-629.7) |
| White blood cell (WBC, 109/L) | 5.11±16.75 (0.01-217) |
| Lymphocyte (LY, 109/L) | 7.60±21.49 (0-100) |
| Basophil (BA, 109/L) | 0.04±0.18 (0-3.2) |
| Red blood cell count (RBC, 1012/L) | 2.88±0.53 (1.5-4.84) |
| Platelet (PLT, 109/L) | 111.88±91.15 (2-625) |
| Hemoglobin (Hgb, g/L) | 87.30±15.02 (50-148) |
| Hematocrit (HCT, L/L) | 0.26±0.05 (0.15-0.46) |
| Busulfan-based dosing regimen |  |
| 2 days | 27 (21%) |
| 3 days | 38 (30%) |
| 4 days | 63 (49%) |

a,The daily dose of the combined drugs used on the day of busulfan plasma samples collection; SD, standard deviation.

**Table S4. GST genetic frequencies**

| **Genotype and detection site** | **SNP** | **N (%)** | **Hardy-Weinberg (*P*)** |
| --- | --- | --- | --- |
| *GSTA1-52 (rs3957356)* | CC | 96 (76.2%) | 0.51 |
|  | TC | 27 (21.4%) |  |
|  | TT | 3 (2.4%) |  |
| *GSTA1-69 (rs3957357)* | AA | 3 (2.4%) | 0.51 |
|  | AG | 27 (21.4%) |  |
|  | GG | 96 (76.2%) |  |
| *GSTA1-513 (rs11964968)* | CC | 3 (2.4%) | 0.51 |
|  | TC | 27 (21.4%) |  |
|  | TT | 96 (76.2%) |  |
| *GSTA1-631 (rs4715333)* | AA | 30 (23.8%) | 0.99 |
|  | CA | 63 (50.0%) |  |
|  | CC | 33 (26.2%) |  |
| *GSTA1-1142 (rs58912740)* | CC | 3 (2.4%) | 0.51 |
|  | GC | 27 (21.4%) |  |
|  | GG | 96 (76.2%) |  |
| *GSTP1-313 (rs1695)* | AA | 82 (65.1%) | 0.89 |
|  | AG | 39 (30.9%) |  |
|  | GG | 5 (4.0%) |  |

GST, glutathione S-transferase enzyme; SNP, single nucleotide polymorphism.

**Table S5. Covariates screening process**

|  | **Model** | **Model Description** | **OFV** | **ΔOFV** | ***df*** | ***P*** |
| --- | --- | --- | --- | --- | --- | --- |
| Forward inclusion | 1 | Basic model **(**Model III) | -1307.41 |  |  |  |
|  | 2 | Add UREA on CL in model 1 | -1331.05 | -23.64 | 1 | <0.01 |
|  | 3 | Add UA on CL in model 2 | -1339.53 | -8.47 | 1 | <0.01 |
|  | 4 | Add LY on CL in model 3 | -1345.67 | -6.14 | 1 | >0.01 |
|  | 5 | Add WBC on CL in model 3 | -1349.19 | -9.67 | 1 | <0.01 |
|  | 6 | Add PHE on CL in model 5 | -1353.83 | -4.63 | 1 | >0.01 |
|  | 7 | Add GGT on V in model 5 | -1365.03 | -15.84 | 1 | <0.01 |
|  | 8 | Add IBIL on CL in model 7 | -1367.50 | -2.47 | 1 | >0.01 |
|  | 9 | Add TBIL on CL in model 7 | -1367.46 | -2.43 | 1 | >0.01 |
|  | 10 | Add MET on CL in model 7 | -1373.57 | -8.54 | 1 | <0.01 |
|  | 11 | Add PHE on V in model 10 | -1380.00 | -6.43 | 1 | >0.01 |
|  | 12 | Add TP on CL in model 10 | -1375.56 | -1.99 | 1 | >0.01 |
|  | 13 | Add DBIL on CL in model 10 | -1374.63 | -1.06 | 1 | >0.01 |
|  | 14 | Add GLB on CL in model 10 | -1377.79 | -4.22 | 1 | >0.01 |
|  | 15 | Add DBIL on V in model 10 | -1375.46 | -1.89 | 1 | >0.01 |
|  | 16 | Add GGT on CL in model 10 | -1376.59 | -3.02 | 1 | >0.01 |
|  | 17 | Add WBC on V in model 10 | -1379.76 | -6.20 | 1 | >0.01 |
|  | 18 | Add ALB on CL in model 10 | -1373.57 | -0.01 | 1 | >0.01 |
|  | 19 | Add TBIL on V in model 10 | -1376.22 | -2.65 | 1 | >0.01 |
|  | 20 | Add CL_BOV in model 10 | -1374.48 | -0.91 | 1 | >0.01 |
|  | 21 | Add V_BOV in model 10 | -1374.95 | -1.38 | 1 | >0.01 |
| Backward elimination | 22 | Remove MET on CL in model 10 | -1365.03 | 8.54 | 1 | >0.001 |
|  | 23 | Remove GGT on V in model 22 | -1349.19 | 15.84 | 1 | <0.001 |
|  | 24 | Remove WBC on CL in model 22 | -1354.31 | 10.72 | 1 | >0.001 |
|  | 25 | Remove UA on CL in model 24 | -1343.48 | 10.82 | 1 | >0.001 |
|  | 26 | Remove UREA on CL in model 25 | -1319.92 | 23.56 | 1 | <0.001 |

OFV, objective function value.

**Table S6.** **Patients’ information with the events**

| **No.** | **Sex** | **Age (years)** | **Weight (kg)** | **Diagnose** | **Busulfan AUC (μM ×min)** | **Transplant type** | **By donor relationship** | **Event-free survival days** | **Events** |
| --- | --- | --- | --- | --- | --- | --- | --- | --- | --- |
| 1 | Male | 3.8 | 16 | AML | 691.47 | CB | Mismatched unrelated donor | 22 | The first graft failure; Second transplant |
| 2 | Male | 2.0 | 11 | WAS | 722.36 | CB | Mismatched unrelated donor | 10 | Death (ICH, MODS) |
| 3 | Female | 0.8 | 8 | AML | 751.01 | CB | Mismatched unrelated donor | 57 | Disease relapse |
| 4 | Male | 0.9 | 8.8 | ALL | 851.33 | BM | Mismatched related donor(father) | 123 | Relapse-related death |
| 5 | Male | 1.4 | 10 | AML | 1173.59 | CB | Mismatched unrelated donor | 23 | Disease relapse |
| 6 | Female | 11.7 | 43 | ALL | 1384.85 | CB | Mismatched unrelated donor | 6 | Death (respiratory and heart failure) |
| 7 | Male | 2.2 | 12 | ALL | 1427.04 | CB | Mismatched unrelated donor | 20 | Death (severe pneumonia) |
| 8 | Male | 7.2 | 19.6 | ALL | 1455.66 | CB | Mismatched unrelated donor | 338 | Death (respiratory failure) |
| 9 | Male | 11.3 | 44 | AML | 1497.73 | BM+PB | Mismatched related donor (father) | 33 | Graft failure--related death |
| 10 | Female | 3.6 | 11.5 | AML | 1514.93 | BM+PB | Mismatched related donor (father) | 27 | Death (severe pneumonia, respiratory failure) |
| 11 | Male | 10.6 | 32.5 | ALL | 1518.61 | BM+PB | Mismatched related donor (father) | 158 | Relapse-related death |
| 12 | Male | 11.6 | 29 | ALL | 1632.72 | BM+PB | Mismatched related donor (father) | 129 | Disease relapse |
| 13 | Female | 5.1 | 17 | ALL | 1643.05 | BM+PB | Mismatched related donor (father) | 356 | Disease relapse |
| 14 | Male | 1.9 | 14.8 | AML | 1797.93 | PB | Mismatched related donor (father) | 43 | Relapse-related death |
| 15 | Male | 16.9 | 63 | AML | 1877.61 | PB | Matched related donor (sister) | 524 | Death (respiratory failure, MODS) |
| 16 | Male | 2.3 | 13.5 | AML | 2155.96 | CB | Mismatched unrelated donor | 26 | Relapse-related death |

ICH, Intracranial hemorrhage; MODS, multiple organ dysfunction syndrome.

**REFERENCES**

Anderson, B.J., and Holford, N.H. (2009). Mechanistic basis of using body size and maturation to predict clearance in humans. *Drug Metab Pharmacokinet* 24(1)**,** 25-36. doi: 10.2133/dmpk.24.25.
